# The Impact of Green Exercise on Cardiovascular and Musculoskeletal Health in Middle-Aged and Older Adults: A Scoping Review

**DOI:** 10.3390/ejihpe16050066

**Published:** 2026-05-09

**Authors:** Pablo J. Marcos-Pardo, Adrián Mateo-Orcajada, Rodrigo Gomes de Souza Vale, Raquel Vaquero-Cristóbal

**Affiliations:** 1 Department of Education, Faculty of Educational Sciences, Universidad de Almería, 04120 Almería, Spain; pjmarcos@ual.es; 2 Active Aging, Exercise, and Health/HEALTHY-AGE Network, Consejo Superior de Deportes, 28040 Madrid, Spain; rodrigogsvale@gmail.com (R.G.d.S.V.); raquel.vaquero@um.es (R.V.-C.); 3 Research Group Movement Sciences and Sport (MS&SPORT), Department of Physical Activity and Sport, Faculty of Sport Sciences, Universidad de Murcia, San Javier, 30720 Murcia, Spain; 4 Dpto. Ciências da Atividade Física, Universidade do Estado do Rio de Janeiro (UERJ), Rio de Janeiro 20550-013, Brazil

**Keywords:** green exercise, outdoor physical activity, cardiovascular health, musculoskeletal health, adults, older adults, natural environments

## Abstract

Growing urbanization has significantly contributed to the global burden of cardiovascular and musculoskeletal diseases. Green exercise, defined as structured physical activity in natural environments, has emerged as a potential intervention to address these health outcomes. This scoping review aims to synthesize the current scientific evidence on the cardiovascular and musculoskeletal benefits of green exercise among middle-aged (35–64 years) and older adults (≥65 years), examining potential mechanisms, intervention characteristics, and practical implications. A systematic search was conducted across PubMed, Web of Science, Scopus, and Google Scholar databases. Methodological quality was assessed to contextualize the strength of the findings. The available evidence suggests potential cardiovascular benefits, particularly in blood pressure regulation and heart rate variability, supported by both high and moderate-quality studies. Regarding musculoskeletal health, high-quality evidence appears to show improvements in muscle strength, lean mass and functional capacity, as well as reductions in fat mass. While green exercise appears to offer additional psychophysiological advantages over indoor exercise, results remain mixed regarding the clinical superiority of natural versus urban outdoor settings. Notably, significant benefits were observed across diverse frequencies and durations, suggesting that a strictly optimal dose–response remains elusive due to study heterogeneity. Consequently, green exercise appears to be a promising, evidence-informed strategy for healthy aging. While it is associated with improvements in cardiovascular and musculoskeletal health, its added value over traditional settings should be viewed as a complementary factor for adherence. Future research should prioritize high-quality mechanistic trials and standardized protocols to refine clinical prescriptions.

## 1. Introduction

The global health landscape is increasingly dominated by lifestyle-related chronic conditions. Cardiovascular disease (CVD) and musculoskeletal disorders remain leading causes of mortality and morbidity worldwide (World Health Organization, 2022). Conditions such as hypertension, diabetes, sarcopenia, and osteoporosis are highly prevalent among older adults (Asavamongkolkul et al., 2024; Jan et al., 2024; Zhang et al., 2025), significantly driving functional decline and poor clinical prognoses (Aïdoud et al., 2023). Paradoxically, rapid urbanization has restricted access to natural environments while exacerbating sedentary behaviors (Hartig et al., 2014; Kondo et al., 2018). While traditional health promotion has historically focused on indoor facilities, emerging evidence suggests that the exercise environment itself is a critical determinant of health outcomes (Gladwell et al., 2013; Thompson Coon et al., 2011).

In this context, outdoor exercise, and more specifically green exercise, defined as structured or purposeful physical activity performed in natural outdoor settings (e.g., parks, forests, coastal areas), where both physical activity and exposure to nature occur simultaneously (Barton & Pretty, 2010; Pretty et al., 2005), represents a paradigm shift that capitalizes on the synergy between physical exertion and nature exposure (Barton & Pretty, 2010; Pretty et al., 2005). Grounded in Attention Restoration Theory (Kaplan, 1995) and Stress Reduction Theory (Ulrich et al., 1991), green exercise provides a therapeutic framework that reduces mental fatigue and physiological stress, potentially optimizing health outcomes beyond those achieved through indoor exercise (Bowler et al., 2010; Hartig et al., 2014).

Regarding cardiovascular health, green exercise may overcome the limitations of traditional interventions by enhancing motivation through esthetic and social engagement (Calogiuri & Chroni, 2014; Mitchell, 2013). Its benefits are mediated through both direct physiological pathways (e.g., improved fitness and blood pressure) and indirect mechanisms related to stress reduction and autonomic regulation (Q. Li, 2010; Maller et al., 2006; Mitchell, 2013). Furthermore, environmental factors such as improved air quality and sunlight exposure may further augment these cardioprotective effects (Q. Li et al., 2008).

From a musculoskeletal perspective, the age-related decline in physical function is a major public health priority (Cruz-Jentoft et al., 2019). Natural environments offer unique biomechanical advantages; for instance, uneven terrain and variable surfaces necessitate multi-planar movements that challenge balance, proprioception, and coordination more dynamically than flat, indoor surfaces (Giles-Corti et al., 2005; Calogiuri et al., 2015a; Niedermeier et al., 2017). Additionally, outdoor exposure facilitates vitamin D synthesis, which is essential for bone health and muscle function in aging populations (Girgis et al., 2013; Holick, 2007).

Despite the growing interest in nature-based interventions, the specific cardiovascular and musculoskeletal benefits of green exercise remain incompletely characterized. Previous reviews have often focused on broad psychological outcomes or heterogeneous populations, limiting the ability to derive targeted clinical recommendations for these specific health domains (Bowler et al., 2010; Gladwell et al., 2013; Thompson Coon et al., 2011).

To address this gap, the present scoping review aims to systematically examine the cardiovascular and musculoskeletal health benefits of green exercise among middle-aged and older adults. Specifically, the objectives are: (a) to synthesize the current evidence on cardiovascular outcomes; (b) to evaluate musculoskeletal adaptations; (c) to examine dose–response relationships and optimal intervention characteristics; and (d) to compare the effects of green exercise with those of traditional indoor exercise. By integrating these two critical health domains within a single framework, this study provides a comprehensive perspective on the role of natural environments in contemporary health promotion.

## 2. Materials and Methods

### 2.1. Study Design and Search Strategy

This scoping review was conducted in accordance with the methodological framework proposed by Arksey and O’Malley (2005) and further refined by the Joanna Briggs Institute (Peters et al., 2020). The reporting adheres to the PRISMA-ScR (Preferred Reporting Items for Systematic Reviews and Meta-Analyses extension for Scoping Reviews) guidelines (Tricco et al., 2018) to ensure transparency and methodological rigor (Supplementary Material S1).

To organize and interpret the findings, a narrative synthesis approach was adopted (Popay et al., 2006), without altering the underlying scoping review methodology. This approach was selected to accommodate the significant heterogeneity in study designs, which precluded meta-analytic synthesis.

The review was retrospectively registered in the Open Science Framework (OSF; https://osf.io/9csab/, accessed on 31 March 2026), to promote transparency and reproducibility (https://doi.org/10.17605/OSF.IO/9CSAB, accessed on 31 March 2026). While retrospective registration is a limitation, it was performed to document the process and minimize post hoc methodological deviations.

The systematic search was concluded on 29 January 2026, across PubMed/MEDLINE, Web of Science, Scopus, and Google Scholar. The search strategy integrated three concept groups (intervention, outcomes, design and population) using Boolean operators: (“green exercise” OR “outdoor physical activity” OR “nature-based exercise” OR “exercise in green spaces” OR “park-based exercise” OR “forest exercise” OR “outdoor fitness” OR “green gym” OR “outdoor training”) AND (“cardiovascular health” OR “cardiovascular fitness” OR “cardiorespiratory fitness” OR “blood pressure” OR “heart rate” OR “musculoskeletal health” OR “muscle strength” OR “bone health” OR “balance” OR “functional capacity” OR “physical fitness” OR “motor function”) AND (“randomized controlled trial” OR “RCT” OR “quasi-experimental” OR “cohort study” OR “cross-sectional” OR “observational”) AND (“adults” OR “elderly” OR “older adults” OR “middle-aged” OR “aging” OR “seniors”) NOT (“children” OR “adolescents” OR “pediatric”). Indoor exercise terms were not excluded to avoid omitting comparative studies (e.g., outdoor vs. indoor).

In addition to searching databases, reference list screening was performed to identify potentially relevant comparative studies not captured in the initial search (*n* = 43). Due to the platform limitations of Google Scholar, searches were conducted using simplified keyword combinations, screening the first 200 results per query according to established recommendations for systematic searches.

No date restrictions were applied, and eligibility was limited to studies in English, Spanish, and Portuguese. The full search strategies for each database are provided in Supplementary Material S2.

### 2.2. Inclusion and Exclusion Criteria

Middle-aged adults were defined as individuals aged 35–64 years, while older adults were defined as those aged ≥ 65 years. These age cut-off points were established because they represent the life phases of the adult life span according to previous research (Franssen et al., 2020; Lachman, 2004). Studies with samples falling clearly outside these ranges (based on mean age or range) were excluded. Inclusion criteria comprised peer-reviewed empirical studies involving middle-aged or older adults engaged in structured physical activity in natural outdoor environments. Eligible designs included randomized controlled trials (RCTs), quasi-experimental, cohort, and cross-sectional studies assessing at least one cardiovascular or musculoskeletal outcome. Only interventions explicitly describing outdoor or nature-based physical activity were categorized as green exercise interventions.

### 2.3. Study Selection Process

**Figure 1:**
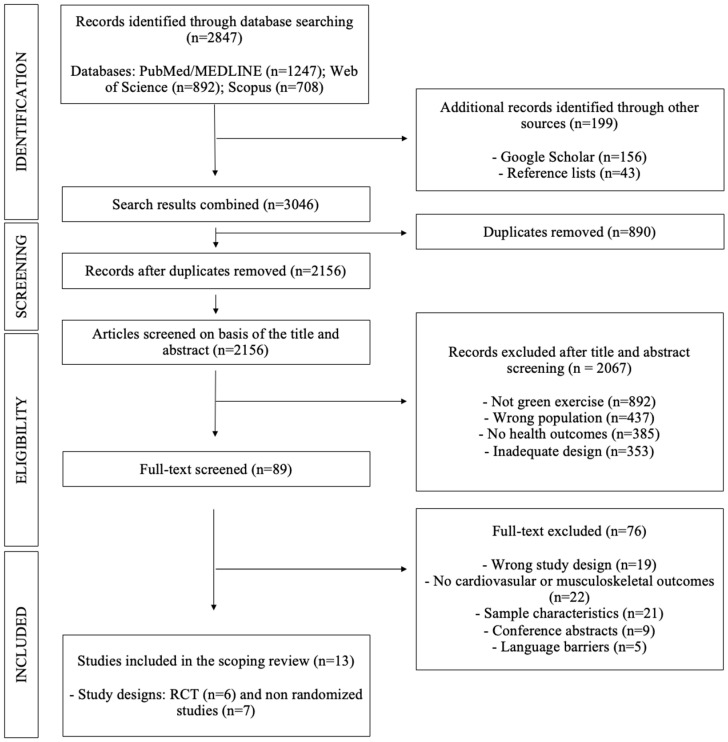
PRISMA diagram of study selection.

Selection followed a two-stage screening process to minimize bias (Higgins et al., 2011, 2024; T. Li et al., 2021; Page et al., 2021). Of the 3046 identified records, 2156 were screened after duplicate removal. Titles and abstracts were independently screened by two reviewers (P.J.M-P and A.M-O), followed by full-text assessment. Study selection was performed independently by two reviewers. Inter-rater reliability was assessed using Cohen’s kappa (*κ*). Agreement was substantial for the initial screening (*κ* = 0.79) and almost perfect for full-text assessment (*κ* = 0.93). Discrepancies were resolved through consensus with a third reviewer (R.V-C). Thirteen studies were finally included in the review. The process was documented using a PRISMA-style flow diagram (Figure 1).

### 2.4. Data Extraction and Quality Assessment

Data were extracted using a standardized form covering authors, design, sample characteristics, study population, intervention (type, frequency, intensity, duration), setting and main results (cardiovascular or musculoskeletal).

Methodological quality and risk of bias were assessed independently by two reviewers (P.J.M-P and A.M-O). The Cochrane Risk of Bias tool (RoB 2) was used for RCTs (Higgins et al., 2011), while the Newcastle-Ottawa Scale (NOS) was employed for non-randomized interventions and observational studies (Wells et al., 2000). Inter-rater reliability was almost perfect for RCTs (*κ* = 0.84) and substantial for non-randomized studies (*κ* = 0.73). Discrepancies were resolved through consensus with a third reviewer (R.G.d.S.V).

The quality assessment of the included primary empirical studies indicated generally moderate-to-high methodological quality (Supplementary Material S3). However, certain inherent limitations must be acknowledged. Due to the nature of outdoor physical activity interventions, blinding of participants and instructors was not feasible, introducing an unavoidable risk of performance bias. Additionally, some outcomes relied on self-reported measures, which may be susceptible to recall or social desirability bias. Quality assessment results were not used as exclusion criteria but served to contextualize the findings. Consequently, greater weight was afforded to evidence from higher-quality studies, while results from moderate or lower-quality studies were interpreted with increased caution.

Furthermore, a qualitative appraisal of the overall strength of evidence was conducted based on consistency of findings and methodological rigor. A formal GRADE approach was not applied, as the primary aim of this study was to map the available evidence rather than to produce pooled effect estimates. Detailed quality scores and individual risk-of-bias assessments are provided in Supplementary Material S3.

### 2.5. Data Synthesis and Analysis

Data synthesis followed the narrative synthesis framework proposed by Popay et al. (2006) for narrative synthesis. Extracted data were systematically coded according to a priori categories (cardiovascular outcomes, musculoskeletal outcomes, intervention characteristics, and comparative effects) which were iteratively refined. An inductive thematic approach was used, allowing specific sub-themes to emerge from the data. Two reviewers independently performed the initial coding, with themes subsequently refined through iterative discussion. Analysis was conducted manually, and inconsistencies across studies were examined in relation to study design, population characteristics, and intervention variability. In cases of conflicting findings, greater interpretative weight was assigned to studies with higher methodological quality and more rigorous designs. The synthesis was structured thematically around cardiovascular and musculoskeletal outcomes, with considerations for participant characteristics (age groups, health status), intervention modalities (aerobic, resistance, combined), and study quality. Results were presented narratively and supported by tables summarizing key study characteristics and findings.

## 3. Results

### 3.1. Study and Participant Characteristics

The included studies were conducted across Europe, North America, and Asia, and comprised RCTs (46.2%) and non-randomized intervention and experimental studies (53.8%). Sample sizes varied considerably across studies. The total participant pool (*n* = 1174) ranged in age from 45.1 to 82.3 years, consistent with the predefined inclusion criteria for middle-aged and older adults. Half of the studies (*n* = 6; 46.2%) focused on middle-aged adults, while the remaining half (*n* = 7; 53.8%) targeted older populations. Most studies included both sexes (*n* = 10; 76.9%). Given this diversity, findings should be interpreted with caution due to the heterogeneity in study designs, intervention characteristics, and outcome measures. A summary of study characteristics is provided in Table 1.

### 3.2. Characteristics of Green Exercise Interventions

There was considerable diversity in the types of green exercise and the settings in which they were performed. Hiking, walking, “forest bathing,” and protocols based on nature walks were the most common modalities (76.9%), followed by multi-component programs combining aerobic and resistance training (30.8%), cycling-based protocols (15.4%) and resistance training with outdoor fitness equipment (7.69%). Settings were equally diverse, including parks and community outdoor spaces (41.7%), forests (41.7%), and mountainous areas (16.7%). Indoor settings (25%) and urban environments (33.3%) were also included. Intervention duration ranged from acute single-session exposures to programs lasting up to 12 months. The review identified: acute protocols consisting of one to three sessions (30.8%); short-term programs lasting 2 to 3 weeks (23.1%); structured programs lasting 8 to 18 weeks (30.8%); and long-term programs lasting 9 months to 1 year (15.4%). Among the intervention studies, the most common session frequency was 2 to 3 times per week (53.8%), although protocols with one session (15.4%) and five sessions per week (15.4%) were also identified. In two studies, weekly frequency was not specified (15.4%). Regarding session duration, the most common range was 45 to 60 min (30.8%), followed by longer protocols (75–120 min) (30.8%). A single study reported 30 min sessions (7.7%), and two studies did not specify duration (15.4%). Supervision levels were generally high across the structured intervention studies.

### 3.3. Cardiovascular Health Outcomes

#### 3.3.1. Blood Pressure, Hemodynamic and Autonomic Response

Two RCTs showed a significant decrease in systolic and diastolic blood pressure (−17.4 mm Hg; −9.2 mm Hg, respectively) following a combined program of aerobic and resistance exercise in a natural setting (Calogiuri et al., 2015a; García-Llorente et al., 2025). These findings are corroborated by non-randomized studies that observed significant blood pressure reductions following walking interventions (de Brito et al., 2020; Q. Li et al., 2011). However, no definitive conclusion can be reached regarding the comparative effectiveness of specific outdoor environments (urban or forest). While one study reported greater reductions in a forest-based group (Q. Li et al., 2011), two others found no significant differences between urban and forest settings (de Brito et al., 2020; Q. Li et al., 2016). Regarding the comparison between indoor and outdoor (park) environments, the only available comparative study indicated that the outdoor group achieved significantly greater reductions (Calogiuri et al., 2015a). In terms of autonomic function, de Brito et al. (2020) reported that exercising in green spaces significantly improved heart rate variability (HRV), specifically increasing parasympathetic activity. Furthermore, natural environments allowed participants to reach heart rate (HR) intensities comparable to indoor settings (Calogiuri et al., 2015b), and forest environments led to a more pronounced reduction in resting HR compared to urban settings (Q. Li et al., 2016).

#### 3.3.2. Cardiorespiratory Fitness and Functional Capacity

Several RCTs have reported improvements in cardiorespiratory fitness. Yıldırım Ayaz et al. (2024) demonstrated gains in the two-minute step test, the 8-foot-up-and-go test, the TUG test, and VO_2_ max; García-Llorente et al. (2025) reported enhanced performance in the 6 m walk test; Zhou et al. (2020) observed functional improvements in both outdoor and indoor groups, although the outdoor group exhibited superior performance in the 10 m walk test and the 2 min walk test; and Marcos-Pardo et al. (2024) demonstrated gains in TUG test using outdoor fitness equipment compared to control group. Among non-randomized studies, Leale et al. (2024) found improved TUG and SPPB scores, while Kono et al. (2004) demonstrated enhanced functional capacity in older adults. Nevertheless, evidence in this domain remains limited by the heterogeneity of study designs and populations.

### 3.4. Musculoskeletal and Body Composition Outcomes

#### 3.4.1. Strength and Balance

Data from multiple intervention studies indicate that outdoor exercise is associated with improvements in muscle strength, balance, and functional ability. For instance, Yıldırım Ayaz et al. (2024) and Leale et al. (2024) observed significant increases in upper- and lower-body strength (chair stand, arm curl, handgrip); and Marcos-Pardo et al. (2024) showed a significant increase in maximal isometric contraction in both legs and arms. Leale et al. (2024) also reported significant improvements in balance performance. Notably, regular outdoor activity was associated with better performance in instrumental activities of daily living (Kono et al., 2004).

#### 3.4.2. Body Composition

Marcos-Pardo et al. (2024) reported a significant decrease in fat mass, as well as an increase in lean mass index. In addition, interventions combining aerobic and resistance training outdoors also showed significant reductions in body fat percentage and visceral fat (García-Llorente et al., 2025). Conversely, non-randomized studies focusing on walking interventions found no significant changes in lipid metabolism or metabolic parameters in older adults (Q. Li et al., 2011, 2016).

### 3.5. Dose–Response and Intervention Characteristics

Programs combining aerobic and strength training appear to offer significant benefits regardless of session frequency or duration (Calogiuri et al., 2015a, 2015b; García-Llorente et al., 2025; Yıldırım Ayaz et al., 2024). Thus, these programs have demonstrated benefits regarding strength, functionality, balance, blood pressure, heart rate, and body composition. Regarding activity type, both walking and cycling yielded significant benefits. Walking remains the most prevalent modality, encouraging higher participation rates and delivering health gains across various intensities, particularly in parks and forests (de Brito et al., 2020; Q. Li et al., 2008, 2011; Reitlo et al., 2018; Zhou et al., 2020). Nevertheless, participant preference also appears critical; for example, cycling combined with resistance training using elastic bands was associated with high levels of enjoyment, intention to persist, and a concurrent reduction in cortisol levels (Calogiuri et al., 2015a, 2015b). However, no definitive conclusion can be drawn regarding optimal frequency or duration. Significant results were observed across both isolated acute sessions and long-term standardized programs, suggesting that the heterogeneity of samples and protocols currently precludes the determination of an optimal dose for natural settings.

### 3.6. Comparison of Effectiveness Between Green Exercise, Urban Outdoor and Indoor Exercise

Comparative studies suggest that both outdoor exercise (in parks, forests, or urban settings) and indoor exercise provide health benefits, although some differences have been observed. In this regard, mixed results were observed: Calogiuri et al. (2015a) and Q. Li et al. (2011) showed that the forest exercise group reduced diastolic blood pressure and cortisol to a greater extent than the indoor group; Reitlo et al. (2018) showed that outdoor walking training was the most common and allowed for more weekly sessions compared to indoor training; Calogiuri et al. (2015b) found that training in nature was associated with a greater intention to exercise in the future compared to indoor training, although changes in HR and RPE were similar; de Brito et al. (2020) indicated that older adults exercising in a park showed significant changes in HRV compared to those exercising in urban environments, although changes in blood pressure were similar in both groups; and Q. Li et al. (2008) found that NK cell activity increased more in the forest group than in the urban group, whereas Q. Li et al. (2016) showed that in the forest group, the reduction in HR was more significant than in the urban group, but there were no differences in blood pressure.

### 3.7. Variability and Inconsistencies Across Studies

Substantial variability was observed in intervention characteristics, population profiles, and outcome measures. Differences in exercise modality, duration, supervision, and environmental context may explain the reported heterogeneity. Some studies reported smaller or non-significant effects, particularly in shorter or less controlled designs. These inconsistencies underscore the need for standardized protocols and rigorous comparative research.

Across the included studies, the most consistently reported quantitative findings included blood pressure reductions, improvements in cardiorespiratory fitness, enhanced HRV in acute and short-term outdoor exercise protocols, and gains in functional performance, including improvements in SPPB scores and fall-related outcomes. Musculoskeletal and body composition adaptations were commonly reflected in increased muscle strength, better balance, improved functional capacity, and reduced fat mass, although the magnitude of these effects varied according to study design, intervention duration, and participant characteristics.

## 4. Discussion

### 4.1. Cardiovascular Findings

The first objective of the present review was to synthesize the current evidence on the impact of green exercise interventions on the cardiovascular health and functional capacity of middle-aged and older adults. Overall, the findings suggest that cardiovascular outcomes associated with green exercise are highly encouraging, with reductions in systolic and diastolic blood pressure (Calogiuri et al., 2015a; García-Llorente et al., 2025). These effects are consistent with the established impact of physical activity on blood pressure regulation (Herrod et al., 2021). Such adaptations are likely mediated by improvements in vasodilation, reductions in peripheral vascular resistance (Green et al., 2017), and decreases in arterial stiffness (Seals et al., 2016). Additionally, the natural environment may synergistically contribute to these effects through the modulation of autonomic function and stress reduction (Q. Li, 2010; Mitchell, 2013). However, these findings should be interpreted cautiously, as some evidence derives from single studies and requires replication.

Regarding autonomic regulation, the observed improvements in HRV and the increase in parasympathetic activity (de Brito et al., 2020) align with known adaptations to aerobic exercise, such as increased vagal tone and improved cardiac efficiency (Casanova-Lizón et al., 2022; Hautala et al., 2003). Interestingly, our results indicate that while exercising in nature allows for reaching intensities (HR) comparable to indoor settings (Calogiuri et al., 2015a), forest environments may be superior to urban outdoor settings in reducing resting HR (Q. Li et al., 2016). This suggests that the multisensory stimulation of natural environments could potentially enhance parasympathetic dominance more effectively than traditional outdoor settings (Gladwell et al., 2013). Nevertheless, the specific mechanisms underlying these effects require further investigation.

In terms of cardiorespiratory fitness and functional capacity, the results demonstrate consistent improvements across several standardized tests (e.g., VO_2_max, 6 min walk test, TUG, and SPPB). These adaptations are supported by physiological mechanisms including increased cardiac output, improved mitochondrial density, and capillarization (Hellsten & Gliemann, 2024; Panzarino et al., 2017). Notably, some evidence in this review suggests that outdoor groups may achieve better performance in functional tests (e.g., 2 min and 10 m walk tests) compared to indoor groups (Zhou et al., 2020). These findings are consistent with previous evidence from indoor interventions (Campbell et al., 2021), yet the outdoor context may offer unique advantages. For instance, the improvement in functional capacity and activities of daily living (Kono et al., 2004; Marcos-Pardo et al., 2024; Yıldırım Ayaz et al., 2024) might be influenced not only by exercise intensity and frequency (Roberts et al., 2017) but also by increased confidence when engaging in outdoor environments (Marselle et al., 2015). Overall, higher levels of physical activity appear to be the key factor underlying improvements in functionality in older adults (Parra-Rizo & Sanchis-Soler, 2020).

However, despite these positive trends, a definitive conclusion on the superiority of specific outdoor environments (e.g., forest vs. urban) cannot yet be drawn due to the inconsistency in comparative studies (de Brito et al., 2020; Q. Li et al., 2016). Furthermore, the evidence regarding functional capacity remains limited by the heterogeneity of study designs and populations. It is also important to note that some conclusions are based on studies of moderate to low methodological quality; therefore, these results should be considered preliminary and interpreted with caution until more rigorous trials confirm this trend.

In summary, the updated evidence suggests that green exercise is an effective strategy for improving cardiovascular health, particularly blood pressure and autonomic regulation, and functional capacity in aging populations, although the specific environmental characteristics that maximize these benefits require further investigation.

### 4.2. Musculoskeletal and Body Composition Findings

The second objective of the review was to evaluate the impact of green exercise on the musculoskeletal system. Overall, the findings indicate that musculoskeletal adaptations associated with green exercise are promising, specifically regarding upper- and lower-body strength (e.g., chair stand, arm curl, handgrip) and balance performance (Leale et al., 2024; Marcos-Pardo et al., 2024; Yıldırım Ayaz et al., 2024). Combined aerobic and resistance training interventions showed the greatest improvements in physical fitness and strength (Yıldırım Ayaz et al., 2024), with additional benefits observed when outdoor programs were supported by telecoaching (Leale et al., 2024). In addition, outdoor resistance training using fitness equipment can elicit significant gains in isometric strength (Marcos-Pardo et al., 2024). These improvements are crucial as muscle strength and power are key determinants of functional independence and fall prevention in older adults (Simpkins & Yang, 2022).

One potential explanation for the positive impact on balance and functional ability is that natural environments, often characterized by uneven surfaces and variable terrain, may promote greater neuromuscular demands and more diverse movement patterns compared to flat, indoor surfaces (Giles-Corti et al., 2005). These environmental characteristics introduce proprioceptive challenges that may enhance postural control and coordination (DaSilva et al., 2021). Furthermore, the association between regular outdoor activity and better performance in instrumental activities of daily living (Kono et al., 2004) suggests that green exercise may facilitate a more effective transfer of training gains to real-world functional tasks.

Regarding body composition, the findings suggest that resistance training and combined aerobic resistance protocols in outdoor settings are associated with significant reductions in body fat and visceral fat, as well as increases in lean mass (García-Llorente et al., 2025; Marcos-Pardo et al., 2024). This is consistent with the established role of combined training in metabolic health. However, it is important to note that these benefits were not observed in interventions based solely on walking (Q. Li et al., 2011, 2016), where no significant changes in lipid metabolism or metabolic parameters were reported. This discrepancy suggests that the type and intensity of the exercise modality may be more decisive for body composition changes than the environment itself.

While outdoor activity may offer additional physiological benefits, such as vitamin D synthesis through sunlight exposure, which supports muscle health (Holick, 2007), the current evidence remains nuanced. Although green exercise promotes improvements in physical fitness, similar gains have been reported in indoor settings (Valenzuela et al., 2023). Therefore, the added value of the “green” component in musculoskeletal health might lie in psychological factors, such as increased confidence and adherence to the program (Marselle et al., 2014), which are essential for long-term functional adaptations.

In summary, current evidence supports the beneficial effects of green exercise on strength, balance, and body composition in middle-aged and older adults. It is important to note that the evidence for improvements in muscle strength and balance is particularly robust, as it is primarily based on high quality studies (Kono et al., 2004; Leale et al., 2024; Marcos-Pardo et al., 2024; Yıldırım Ayaz et al., 2024). In contrast, findings related to body composition and metabolic parameters show more inconsistency; while high quality evidence (García-Llorente et al., 2025) supports fat mass reduction through combined training, the lack of effects in walking programs (Q. Li et al., 2011, 2016) suggests that exercise intensity, rather than the environment, is the primary driver for metabolic changes. This heterogeneity in metabolic responses across different study designs highlights the need for further research to isolate the specific contribution of the natural environment versus the exercise protocol.

### 4.3. Dose–Response Findings

The third objective of the present study was to examine the characteristics of green exercise interventions and potential dose–response relationships. Unlike previous perspectives that suggested a specific optimal volume, our updated findings indicate that green exercise programs, particularly those combining aerobic and resistance training, offer significant benefits across a wide range of frequencies and durations (Calogiuri et al., 2015a; García-Llorente et al., 2025; Yıldırım Ayaz et al., 2024).

The lack of a definitive conclusion regarding an optimal dose stems from the high heterogeneity of the evidence. Significant physiological and functional improvements were observed both in standardized training programs lasting several months and in isolated acute sessions. This suggests that while long-term adaptations are supported by progressive overload, even short-term exposure to exercise in natural settings can trigger immediate positive responses in variables such as blood pressure and cortisol levels (Calogiuri et al., 2015a; Q. Li et al., 2011).

Regarding the type of activity, walking remains the most studied and popular modality, yielding benefits regardless of intensity, especially when performed in parks or forests (de Brito et al., 2020; Zhou et al., 2020). This aligns with the “green exercise” concept, where the environment may lower the perception of effort, encouraging greater participation (Reitlo et al., 2018). However, our results also highlight the importance of participant preference and enjoyment. For instance, cycling combined with resistance training has shown high levels of adherence and intention to continue (Calogiuri et al., 2015b), which is crucial for long-term health maintenance in aging populations.

While traditional physical activity guidelines (Bull et al., 2020) emphasize specific weekly volumes, the evidence in this review suggests a more flexible approach for green exercise. The psychological benefits, such as reduced stress (cortisol), combined with physiological gains (strength, HRV, and body composition), appear to be a robust outcome of the “green” component, potentially mitigating the traditional dose–response ceiling effects observed in indoor settings (Yao et al., 2021).

In summary, although a strictly optimal dose–response remains elusive, the interventions that demonstrated significant physiological adaptations typically utilized session durations ranging from 45 to 90 min, with a weekly frequency of 2 to 3 sessions (Calogiuri et al., 2015a; García-Llorente et al., 2025; Marcos-Pardo et al., 2024; Yıldırım Ayaz et al., 2024). These parameters align with the volume of activity generally required to elicit structural cardiovascular and musculoskeletal changes in aging populations. However, the observation that even shorter, acute sessions (5–30 min) or low-frequency walking programs (de Brito et al., 2020) can significantly impact blood pressure and autonomic function suggests that the minimum effective dose for green exercise may be lower than for indoor settings, likely due to the added psychophysiological benefit of the natural environment. Therefore, while 150 min of moderate-to-vigorous activity per week remains a robust target for overall health, green exercise should be promoted as a flexible and highly accessible modality, where any duration of exposure can contribute to meaningful health outcomes in middle-aged and older adults.

The difficulty in establishing a clear dose–response relationship is exacerbated by the inclusion of studies with disparate methodological quality. While high-quality interventions often employ structured multi-week programs, some of the evidence regarding acute benefits or intentions to practice stems from moderate- or low-quality studies. Therefore, although green exercise appears flexible in its delivery, professionals should prioritize the protocols established in high quality trials (2–3 sessions of combined training) to ensure predictable adaptations. Future research should prioritize more homogeneous protocols to establish precise dose–response relationships for cardiovascular and musculoskeletal health.

### 4.4. Comparison of Effectiveness Between Green Exercise, Urban Outdoor and Indoor Exercise

The fourth objective of the present study was to compare the cardiovascular and musculoskeletal benefits of green exercise with those of traditional indoor exercise. Overall, the evidence suggests that while all modalities provide significant health benefits, the natural environment may offer superior physiological and behavioral advantages in specific domains.

Regarding cardiovascular and endocrine responses, the findings indicate that forest-based exercise may be more effective than indoor or urban settings for reducing diastolic blood pressure and cortisol levels (Calogiuri et al., 2015a; Q. Li et al., 2011). Similarly, natural environments appear to trigger more pronounced changes in autonomic function (HRV) and immune response, specifically NK cell activity, compared to urban outdoor settings (de Brito et al., 2020; Q. Li et al., 2008), although the immunological evidence in this population remains emerging and requires further validation. These findings are consistent with Attention Restoration Theory (Kaplan, 1995) and Stress Reduction Theory (Ulrich et al., 1991), suggesting that natural environments may enhance both physiological and psychological responses to exercise. One potential mechanism underlying improvements in HRV and blood pressure is the influence of natural environments on perceived stress, autonomic balance, and affective regulation. From a psychological perspective, green exercise has been associated with increased positive affect and reduced perceived stress, which may reinforce motivation and long-term adherence (Barton & Pretty, 2010).

From a behavioral and psychological perspective, the natural environment seems to act as a catalyst for long-term adherence. Although physiological markers during exercise, such as heart rate and perceived exertion, may be similar between indoor and outdoor settings (Calogiuri et al., 2015b), green exercise is associated with a greater intention to exercise in the future and a higher frequency of weekly sessions (Calogiuri et al., 2015b; Reitlo et al., 2018). This supports the Attention Restoration Theory (Kaplan, 1995), which posits that natural settings reduce mental fatigue and make physical activity feel more intrinsically rewarding and less cognitively demanding.

In conclusion, while the current evidence points toward a potentially favorable role of natural environments in enhancing certain psychophysiological responses, these findings must be interpreted with caution. The limited number of direct comparative studies and the presence of mixed results regarding hemodynamic variables (e.g., blood pressure) preclude any definitive claim about the general superiority of green exercise over urban or indoor modalities. Rather than a replacement for traditional settings, green exercise should be viewed as a promising complementary strategy that may facilitate higher levels of enjoyment and intention to practice. Future research with more standardized protocols and larger samples is strictly necessary to determine whether the natural environment provides a significant added value beyond the well-known benefits of physical activity itself.

### 4.5. Clinical and Public Health Implications

The emerging evidence suggests that green exercise holds potential as an evidence-informed strategy for cardiovascular and musculoskeletal health promotion. While a definitive dose–response remains to be established, exercise prescriptions could effectively follow the FITT-VP framework adapted to natural environments, prioritizing the combined aerobic and resistance protocols (2–3 times weekly) that demonstrated robust results in high-quality trials. For musculoskeletal health, incorporating balance challenges through terrain variability is particularly relevant for functional aging.

At a population level, investment in accessible green infrastructure represents a potentially cost-effective public health strategy. Social prescribing models, where healthcare providers refer patients to nature-based activities, show promise as a way to bridge clinical and community care while reducing healthcare costs. Green exercise is not only effective for individual health but also aligns with environmental sustainability. Park-based programs may offer lower environmental impact than gym-based alternatives (Jansson et al., 2019) and, if properly designed, support biodiversity and mitigate urban heat islands.

However, effective implementation depends on addressing safety and accessibility. Beyond risk assessment protocols for terrain and weather, training for exercise professionals in outdoor leadership is essential. Such expertise is crucial not only for safety but also for fostering participant confidence and adherence, particularly in older populations who may perceive outdoor environments as more challenging.

### 4.6. Limitations and Future Research

Despite strong evidence, several limitations should be noted. First, the high heterogeneity in study designs, varying in intervention type, duration, and outcomes, precludes a meta-analysis and complicates direct comparisons. Second, while the methodological quality was generally moderate-to-high, the reliance on small sample sizes and the inherent difficulty of participant blinding in environmental interventions reduce the certainty of some conclusions, particularly regarding the specific added value of green exercise over indoor settings. Third, it remains difficult to determine whether the observed benefits are primarily driven by the exercise load or by extrinsic factors such as social interaction and enhanced motivation, which are inherently linked to outdoor group activities. Fourth, the retrospective registration of this review in the Open Science Framework is a limitation compared to prospective registration; however, this was done to ensure transparency and adherence to open science principles, and no substantial deviations from the protocol occurred.

Future research should prioritize head-to-head mechanistic trials comparing identical exercise protocols in indoor and outdoor settings to isolate the environmental effect. Systematic dose–response studies and the integration of wearable technology are needed to objective and personalize load monitoring. Finally, economic assessments and research focused on environmental equity are essential to address barriers such as safety and accessibility in underserved populations.

## 5. Conclusions

This scoping review indicates that green exercise is a promising and multifaceted strategy for improving cardiovascular health, muscular strength and functional capacity in middle-aged and older adults. While the findings suggest encouraging improvements in blood pressure regulation, HRV, and balance, these should be viewed as preliminary due to the heterogeneity of the interventions and the limited number of direct indoor-outdoor comparative studies. The synergistic interaction between physical activity and natural environments appears to facilitate not only physiological adaptations but also psychological benefits that may enhance adherence.

Regarding training parameters, although a strictly optimal dose–response remains elusive due to the diversity of the evidence, significant benefits have been observed across various frequencies and durations, with combined aerobic and resistance protocols showing the most robust results in high-quality trials. However, it remains challenging to fully disentangle the independent effect of the natural setting from exercise dose and behavioral confounding factors.

From a public health perspective, green exercise represents a cost-effective and sustainable intervention that aligns with the goals of healthy aging. Rather than a replacement for traditional exercise, it should be promoted as a valuable complementary modality that may lower the threshold for physical activity participation through increased enjoyment. Future research must prioritize high-quality mechanistic trials and standardized protocols to refine prescription guidelines and ensure equitable access to nature-based health promotion.

As global urbanization increases, integrating green exercise into clinical and community practice offers a strategic approach to maintain population health while supporting environmental sustainability.

## Figures and Tables

**Table 1:** Characteristics of included studies.

Nº	Authors	Study Design	Sample	Population	Intervention	Setting	Main Results
**SECTION A—Randomized Controlled Trials (*n* = 6)**							
1	Yıldırım Ayaz et al. (2024)	Multicenter RCT	*n* = 90 Age: 74.0 ± 6.4 yr	Older adults, Turkey	Aerobic (AE), aerobic + resistance (AE + RE) green exercise and control. 12 wk; 1×/wk; 50 min/session.	Outdoor park/green space	Both groups improved (AE and AE + RE): chair stand, arm curl, two-minutes step, chair sit and reach, back scratch, 8-foot-up and go, VO_2_ max., TUG and handgrip muscle strength (*p* < 0.05).
2	García-Llorente et al. (2025)	Randomized Controlled Trial (ACTIVA-Senior Study)	*n* = 46 Age: 66.0 ± 5.1 yr	Community-dwelling older adults, Spain	Combined aerobic + resistance outdoor exercise. 18 wk; 2×/wk; 60 min/session.	Local parks and outdoor fitness areas (outdoor)	Systolic blood pressure (−17.4 mmHg, *p* < 0.001), diastolic blood pressure (−9.2 mmHg, *p* < 0.001), 6MWT (+64.7 m, *p* < 0.001), percent body fat (−1.3%; *p* = 0.007), visceral fat level (−0.9; *p* = 0.002)
3	Zhou et al. (2020)	Randomized Controlled Trial	*n* = 22 Age: 80.2 ± 3.7 yr	Local nursing home, China	Outdoor multisurface terrain (OMTG) vs. indoor solid ground (ISGG). 3 wk; 5×/wk; 30 min/session.	Outdoor multisurface terrain vs. indoor solid ground	Both groups improved functional capacity (*p* < 0.05). However, OMTG showed greater improvement in 10 mWT (*p* = 0.049), MTWT (*p* = 0.020) and 2 MWT (*p* < 0.001)
4	Calogiuri et al. (2015a)	Pilot RCT (workplace intervention)	*n* = 14 Age: 49.0 ± 8.0 yr	Office workers, Norway	Green exercise vs. indoor exercise 2 wk; 2×/wk; 45 min (25 min of biking session and 20 min of strength session using elastic rubber bands).	Forest area vs. indoor setting (gym-hall)	Nature group reduced diastolic blood pressure (*p* = 0.05) and cortisol (*p* = 0.04).
5	Reitlo et al. (2018)	Randomized Controlled Trial (Generation 100)	*n* = 618 Age: 72.4 ± 2.0 yr	Community-dwelling older adults, Norway	Participants completed exercise logs after each exercise session they performed for one year. They were randomly assigned to MCT or HIIT.	Outdoor and indoor exercise settings, Norway	Outdoor was the most common exercise location in both training groups. Walking was the most common type in both groups, but MCT had a higher proportion of sessions than HIIT (*p* < 0.001).
6	Marcos-Pardo et al. (2024)	Randomized Controlled Trial	*n* = 128 Age: 59.0 ± 7.1 yr	Middle-aged and older adults, Spain	8 wk; 2×/wk; 45–60 min. Resistance training using outdoor fitness equipment.	Outdoor fitness equipment vs. control group (regular daily activities)	Outdoor group increased lean mass index (*p* = 0.002), maximal isometric contraction in both legs (*p* < 0.001) and arms (*p* < 0.001). In addition, a greater decrease in fat mass (*p* < 0.001) and TUG time (*p* < 0.001) were found in the outdoor group compared to control.
**SECTION B—Non-Randomized Intervention and Observational Studies (*n* = 7)**							
7	Leale et al. (2024)	Supervised outdoor exercise combined with tele coaching (Quasi experimental design)	*n* = 60 Age: 71.2 ± 6.0 yr	Older adults, Italy	Supervised outdoor exercise + tele coaching vs. untrained group 8 wk; 5×/wk (2 involved supervised outdoor exercise (90 min) and 3 involved tele coaching (40 min)).	Outdoor urban park	Handgrip strength (*p* < 0.001), TUG (*p* < 0.001), SPPB (*p* = 0.012), and Tinetti scale (*p* = 0.002).
8	Calogiuri et al. (2015b)	Non-Randomized trial	*n* = 14 Age: 48.5 ± 7.3 yr	Healthy adults, Norway	Outdoor vs. indoor exercise Two sessions (25 min biking and 50 min strength using elastic resistance rubber bands).	Natural area vs. indoor (gym-hall)	Similar HR and RPE in both environments. Nature group reported higher enjoyment (*p* = 0.02) and intention to exercise in the future (*p* < 0.001).
9	de Brito et al. (2020)	Non-Randomized Crossover Study	*n* = 23 Age: 49.7 ± 6.5 yr	Middle aged adults, USA	Green walking intervention followed by suburban walking 3 wk per intervention with 2 wk washout period between them. Weekly 50 min walking sessions for each intervention.	Urban park vs. urban street	Higher mean HRV and less HRV reduction during green walking compared to suburban walking (*p* < 0.001). Systolic and diastolic blood pressure decreased for both green and suburban walking (*p* < 0.003).
10	Q. Li et al. (2008)	Non-Randomized Crossover Study	*n* = 12 Age: 45.1 ± 6.7 yr	Healthy middle-aged male adults, Japan	Forest bathing trip (walking in a forest for 3 days and stayed for 2 nights at a nearby hotel within the forest) vs. city tourist visit (walking in a tourist route for 3 days and stayed for 2 nights at a hotel in the city center).	Forest vs. urban city environment	The forest trip increased human NK cell activity (*p* < 0.001) and decreased adrenaline concentration (*p* < 0.001).
11	Q. Li et al. (2011)	Controlled Experimental Study	*n* = 16 Age: 57.4 ± 11.6 yr	Healthy middle-aged male adults, Japan	Acute forest walking vs. urban walking. Single session; 4 h; moderate intensity. One week between sessions.	Forest environment and urban environment	Systolic and diastolic blood pressure levels were lower in the forest (*p* < 0.01). No significant differences were found on lipid metabolism nor sleep duration.
12	Q. Li et al. (2016)	Controlled Experimental Study	*n* = 19 Age: 51.2 ± 8.8 yr	Middle-aged males, Japan	Walk in urban area (1 session, 80 min) and walk in forest area (1 session, 80 min). One week between sessions.	Forest environment and urban environment	Forest trip significantly reduced the subjects’ HR (*p* < 0.01). No significant difference in blood pressure between forest and urban areas. No effects on metabolic parameters.
13	Kono et al. (2004)	Prospective Cohort study	*n* = 112 Age: 82.3 ± 7.1 yr	Frail older adults living at home, Japan	Frequency of going outdoors over 9 months.	Community outdoor settings	Older adults going outdoors more often being more highly functional (IADL: *p* = 0.002; functional capacity: *p* = 0.006; and instrumental self-maintenance: *p* = 0.007)

Legend: RCT = Randomized Controlled Trial; HRV = Heart Rate Variability; SPPB = Short Physical Performance Battery; IADL = Instrumental Activities of Daily Living; VO_2_max = maximal oxygen uptake; NK = Natural Killer cells; 10 mWT = 10 m walk test; MTWT = multisurface terrain walk test; 2 MWT = 2 min walk test; HR = Heart Rate; yr = years; min = minutes; wk = weeks.

## Data Availability

No data was used for the research described in the article. Data sharing is not applicable to this article as no new data were created or analyzed in this study.

## Supplementary Materials

The following supporting information can be downloaded at: https://www.mdpi.com/article/10.3390/ejihpe16050066/s1. Supplementary Material S1: Preferred Reporting Items for Systematic Reviews and Meta-Analyses extension for Scoping Reviews; Supplementary Material S2: Full search strategies for all databases, including Boolean operators and applied filters; and Supplementary Material S3: Study-Level Quality Assessment.
