# Supplementary material for: The Impact of Green Exercise on Cardiovascular and Musculoskeletal Health in Middle-Aged and Older Adults: A Scoping Review
Source: Eur J Investig Health Psychol Educ. 2026 May 9;16(5):66. doi: 10.3390/ejihpe16050066 (PMC13206490; doi:10.3390/ejihpe16050066)
Supplement: Supplementary file 1 [file ejihpe-16-00066-s001.zip › Supplementary Material S1.pdf]

Preferred Reporting Items for Systematic reviews and Meta-Analyses extension for Scoping Reviews (PRISMA-ScR) Checklist

Supplementary Material S1

Article: The Impact of Green Exercise on Cardiovascular and Musculoskeletal Health in Middle-Aged Adults and Older Adults: A Scoping Review

Authors: Pablo J. Marcos-Pardo, Adrián Mateo-Orcajada, Rodrigo Gomes de Souza Vale and Raquel Vaquero-Cristóbal

Instructions: This completed checklist maps each PRISMA-ScR item to the current manuscript sections. Page numbers should be finalized after journal typesetting; therefore, the column reports section/table/figure locations and editorial notes where useful.

SECTION	ITEM	PRISMA-ScR CHECKLIST ITEM	REPORTED LOCATION / PAGE #	STATUS
**TITLE**	Title<br>1	Identify the report as a scoping review.	Title page	Yes
**ABSTRACT**	Structured summary<br>2	Provide a structured summary that includes, as applicable: background, objectives, eligibility criteria, sources of evidence, charting methods, results, and conclusions that relate to the review questions and objectives.	Abstract	Yes
**INTRODUCTION**	Rationale<br>3	Describe the rationale for the review in the context of what is already known. Explain why the review questions/objectives lend themselves to a scoping review approach.	Introduction, paragraphs 1-5	Yes
INTRODUCTION	Objectives<br>4	Provide an explicit statement of the questions and objectives being addressed with reference to key elements such as population or participants, concepts, and context.	Introduction, final paragraph	Yes
**METHODS**	Protocol and registration<br>5	Indicate whether a review protocol exists; state if and where it can be accessed, and if available, provide registration information.	Methods 2.1	Yes
METHODS	Eligibility criteria<br>6	Specify characteristics of the sources of evidence used as eligibility criteria, such as years considered, language, and publication status, and provide a rationale.	Methods 2.1 and 2.2	Yes
METHODS	Information sources*<br>7	Describe all information sources in the search, such as databases with dates of coverage and contact with authors to identify additional sources, as well as the date the most recent search was executed.	Methods 2.1	Yes
METHODS	Search<br>8	Present the full electronic search strategy for at least one database, including any limits used, such that it could be repeated.	Methods 2.1 and Supplementary Material S2	Yes
METHODS	Selection of sources of evidence†<br>9	State the process for selecting sources of evidence, including screening and eligibility, included in the scoping review.	Methods 2.3	Yes
METHODS	Data charting process‡<br>10	Describe the methods of charting data from the included sources of evidence, including forms, whether charting was independent or duplicate, and processes for obtaining/confirming data from investigators.	Methods 2.4	Partial
METHODS	Data items<br>11	List and define all variables for which data were sought and any assumptions and simplifications made.	Methods 2.4	Yes
METHODS	Critical appraisal of individual sources of evidence§<br>12	If done, provide a rationale for conducting a critical appraisal of included sources of evidence; describe methods used and how this information was used in data synthesis.	Methods 2.4 and Supplementary Material S3	Yes

SECTION	ITEM	PRISMA-ScR CHECKLIST ITEM	REPORTED LOCATION / PAGE #	STATUS
METHODS	Synthesis of results<br>13	Describe the methods of handling and summarizing the data that were charted.	Methods 2.5	Yes
**RESULTS**	Selection of sources of evidence<br>14	Give numbers of sources of evidence screened, assessed for eligibility, and included, with reasons for exclusions at each stage, ideally using a flow diagram.	Methods 2.3 and Figure 1	Yes
RESULTS	Characteristics of sources of evidence<br>15	For each source of evidence, present characteristics for which data were charted and provide the citations.	Results 3.1 and Table 1	Yes
RESULTS	Critical appraisal within sources of evidence<br>16	If done, present data on critical appraisal of included sources of evidence.	Methods 2.4 and Supplementary Material S3	Yes
RESULTS	Results of individual sources of evidence<br>17	For each included source of evidence, present the relevant data that were charted that relate to the review questions and objectives.	Table 1 and Results 3.3-3.6	Yes
RESULTS	Synthesis of results<br>18	Summarize and/or present the charting results as they relate to the review questions and objectives.	Results 3.2-3.7	Yes
**DISCUSSION**	Summary of evidence<br>19	Summarize the main results, including overview of concepts, themes, and types of evidence available, link to review questions/objectives, and consider relevance to key groups.	Discussion 4.1-4.5	Yes
DISCUSSION	Limitations<br>20	Discuss the limitations of the scoping review process.	Discussion 4.6	Yes
DISCUSSION	Conclusions<br>21	Provide a general interpretation of the results with respect to the review questions/objectives, as well as potential implications and/or next steps.	Conclusions	Yes
**FUNDING**	Funding<br>22	Describe sources of funding for the included sources of evidence, as well as sources of funding for the scoping review. Describe the role of funders of the scoping review.	Funding and Conflicts of Interest	Yes
