# Supplementary material for: The Impact of Green Exercise on Cardiovascular and Musculoskeletal Health in Middle-Aged and Older Adults: A Scoping Review
Source: Eur J Investig Health Psychol Educ. 2026 May 9;16(5):66. doi: 10.3390/ejihpe16050066 (PMC13206490; doi:10.3390/ejihpe16050066)
Supplement: Supplementary file 1 [file ejihpe-16-00066-s001.zip › Supplementary Material S2.pdf]

Supplementary Material S2: Full search strategies for all databases

Overview

The literature search was conducted across four electronic databases: **PubMed/MEDLINE, Web of Science, Scopus, and Google Scholar**. The search strategy combined three main concept groups:

1. green exercise / outdoor physical activity
2. cardiovascular and musculoskeletal outcomes
3. study design and population

Boolean operators (AND, OR, NOT) were used to combine terms. Searches were adapted to the syntax of each database.

No restrictions on publication date were applied. Studies published in English, Spanish, and Portuguese were considered eligible.

1. PubMed/MEDLINE

Searches were performed in title and abstract fields when applicable.

("green exercise" OR "outdoor physical activity" OR "nature-based exercise" OR "exercise in green spaces" OR "park-based exercise" OR "forest exercise" OR "outdoor fitness" OR "green gym" OR "outdoor training") AND ("cardiovascular health" OR "cardiovascular fitness" OR "cardiorespiratory fitness" OR "blood pressure" OR "heart rate" OR "musculoskeletal health" OR "muscle strength" OR "bone health" OR "balance" OR "functional capacity" OR "physical fitness" OR "motor function")AND ("randomized controlled trial" OR "RCT" OR "quasi-experimental" OR "cohort study" OR "cross-sectional" OR "observational") AND ("adults" OR "older adults" OR "elderly" OR "middle-aged" OR "aging" OR "seniors") NOT ("children" OR "adolescents" OR "pediatric")

Filters applied:

- Humans
- Article types: clinical trial, observational study
- Languages: English, Spanish, Portuguese

2. Web of Science (Core Collection)

TS=("green exercise" OR "outdoor physical activity" OR "nature-based exercise" OR "exercise in green spaces" OR "park-based exercise" OR "forest exercise" OR "outdoor fitness" OR "green gym" OR "outdoor training") AND TS=("cardiovascular health" OR "cardiovascular fitness" OR "cardiorespiratory fitness" OR "blood pressure" OR "heart rate" OR "musculoskeletal health" OR "muscle strength" OR "bone health" OR "balance" OR "functional capacity" OR "physical fitness" OR "motor function") AND TS=("randomized controlled trial" OR "RCT" OR "quasi-experimental" OR "cohort study" OR "cross-sectional" OR "observational") AND TS=("adults" OR "older adults" OR "elderly" OR "middle-aged" OR "aging" OR "seniors") NOT TS=("children" OR "adolescents" OR "pediatric")

Filters applied:

- Document types: Article
- Languages: English, Spanish, Portuguese

3. Scopus

TITLE-ABS-KEY("green exercise" OR "outdoor physical activity" OR "nature-based exercise" OR "exercise in green spaces" OR "park-based exercise" OR "forest exercise" OR "outdoor fitness" OR "green gym" OR "outdoor training") AND TITLE-ABS-KEY("cardiovascular health" OR "cardiovascular fitness" OR "cardiorespiratory fitness" OR "blood pressure" OR "heart rate" OR "musculoskeletal health" OR "muscle strength" OR "bone health" OR "balance" OR "functional capacity" OR "physical fitness" OR "motor function") AND TITLE-ABS-KEY("randomized controlled trial" OR "RCT" OR "quasi-experimental" OR "cohort study" OR "cross-sectional" OR "observational") AND TITLE-ABS-KEY("adults" OR "older adults" OR "elderly" OR "middle-aged" OR "aging" OR "seniors") AND NOT TITLE-ABS-KEY("children" OR "adolescents" OR "pediatric")

Filters applied:

- Document type: Article
- Language: English, Spanish, Portuguese

4. Google Scholar

Searches were conducted using combinations of keywords due to platform limitations:

"green exercise" AND cardiovascular AND musculoskeletal AND adults
"outdoor physical activity" AND health AND older adults
"nature-based exercise" AND cardiovascular fitness

The first 200 results per search string were screened, following established recommendations for systematic search approaches in Google Scholar.

Additional Sources

- Reference lists of included studies
- Manual search of key reviews

Notes on search strategy

- Synonyms such as "elderly", "older adults", and "aging" were intentionally included to maximize search sensitivity.
- Search strategies were adapted to each database while maintaining conceptual consistency.

The search process was independently conducted and cross-checked by two reviewers to ensure accuracy and completeness.

Searches were last updated on January 29, 2026.
