# Supplementary material for: The Impact of Green Exercise on Cardiovascular and Musculoskeletal Health in Middle-Aged and Older Adults: A Scoping Review
Source: Eur J Investig Health Psychol Educ. 2026 May 9;16(5):66. doi: 10.3390/ejihpe16050066 (PMC13206490; doi:10.3390/ejihpe16050066)
Supplement: Supplementary file 1 [file ejihpe-16-00066-s001.zip › Supplementary Material S3.pdf]

Supplementary Material S3. Study-Level Quality Assessment of Included Primary Studies

The Impact of Green Exercise on Cardiovascular and Musculoskeletal Health in Middle-Aged Adults and Older Adults: A Scoping Review

This supplementary table presents study-level quality assessments for the primary empirical studies included in this scoping review. Methodological quality was assessed independently by two reviewers, with disagreements resolved by a third reviewer. The Cochrane Risk of Bias 2 (RoB 2) tool was applied to randomized controlled trials (Section A), and the Newcastle-Ottawa Scale (NOS) was applied to non-randomized intervention, experimental, and observational studies (Sections B and C). Systematic reviews, meta-analyses, narrative reviews, integrative reviews, conceptual papers, and contextual references were not subjected to formal quality assessment and are therefore not included in this table. Quality assessment was used to support interpretation of findings and was not applied as a criterion for study exclusion, consistent with scoping review methodology.

Panel A. Risk of Bias Assessment — Randomized Controlled Trials (RoB 2 Tool, n = 6)

■ Low Risk ■ Some Concerns ■ High Risk D1=Randomization · D2=Allocation Concealment · D3=Blinding of Participants/Personnel · D4=Blinding of Outcome Assessment · D5=Incomplete Outcome Data · D6=Selective Reporting

Study (Authors, Year)	D1 Randomiz-ation	D2 Deviation from intended interventions	D3 Missing outcome data	D4 Measurement of the outcome	D5 Selection of the reported result	Overall RoB
Yıldırım Ayaz et al. (2024)	L	L	L	L	L	5/5
García-Llorente et al. (2025)	L	U	L	L	L	5/5
Zhou et al. (2020)	L	H	U	L	L	3/5
Calogiuri, Evensen et al. (2015)	L	H	L	L	L	4/5
Reitlo et al. (2018)	L	H	L	L	L	4/5
Marcos-Pardo et al. (2024)	L	L	L	L	L	5/5
**Domain Summary (% of 6 RCTs)**	**L: 100%<br>U: 0%<br>H: 0%**	**L: 33.3%<br>U: 16.7%<br>H: 50%**	**L: 83.3%<br>U: 16.7%<br>H: 0%**	**L: 100%<br>U: 0%<br>H: 0%**	**L: 100%<br>U: 0%<br>H: 0%**	**L: 50%<br>U: 33.3%<br>H: 16.7%**

Note: L = Low Risk; U = Some Concerns; H = High Risk.

Panel B. Newcastle-Ottawa Scale (NOS) — Non-Randomized Intervention and Observational Studies (n = 7)

■ Excellent (≥ 7★) ■ Good (5–6★) ■ Poor (≤ 4★) Selection max 4★ · Comparability max 2★ · Outcome/Exposure max 3★

Study (Authors, Year)	Selection (max 4 ★)	Comparability (max 2 ★)	Outcome / Exposure (max 3 ★)	Total /9	Quality Judgment
Leale et al. (2024)	★★	★★	★★★	7/9	High
Calogiuri, Nortdug et al. (2015)	★★		★★	4/9	Low
De Brito et al. (2020)	★★	★	★★★	6/9	Moderate
Li et al. (2008)	★★★	★	★★	6/9	Moderate
Li et al. (2011)	★★★	★	★★	6/9	Moderate
Li et al. (2016)	★★★	★★	★★	7/9	High
Kono et al. (2004)	★★★	★★	★★	7/9	High

Panel C. Overall Methodological Quality Summary — Primary Empirical Studies (N = 12)

Study Design	n Studies	High Quality (Low RoB / ≥ 7★)	Moderate Quality (Some Concerns / 5–6★)	Low Quality (High RoB / ≤ 4★)

Randomized Controlled Trials (RoB 2)	6	3 (50.0%)	2 (33.3%)	1 (16.7%)
Non-Randomized Intervention and Observational Studies (NOS)	7	3 (42.9%)	3 (42.9%)	1 (14.2%)
**TOTAL**	**13**	**6 (46.2%)**	**5 (38.5%)**	**2 (15.3%)**

Narrative Quality Assessment Summary

Among the 6 randomized controlled trials assessed with the RoB 2 tool, 3 (50.0%) were rated as high quality (low overall risk of bias), 2 (33.3%) were rated as moderate quality (some concerns) and 1 (16.7%) as low quality (high risk of bias). Randomization procedures (D1), measurement of outcomes (D4) and selection of the reported result (D5) were rated as low risk in all trials (100%). Missing outcome data (D3) was adequate in the majority of studies (5/6). Deviation from intended interventions (D2) was rated as high risk in three RCTs (50%), reflecting the inherent impossibility of concealing outdoor versus indoor exercise conditions from participants, being a structural limitation of the study design rather than a methodological flaw.

Among the 7 non-randomized intervention and observational studies assessed with the NOS, 3 (42.9%) were rated as high quality, 3 (42.9%) were rated as moderate quality and 1 (14.2%) was rated as low quality. Across both non-randomized designs, studies generally demonstrated adequate selection procedures and outcome assessment. The most common limitation was partial control for comparability, reflecting the inherent challenges of confounding in non-randomized designs.

Overall, methodological quality was generally high and moderate (84.7%) across the 13 included primary studies, with 6 (46.2%) rated as high quality and 5 (38.5%) as moderate quality. Only two studies were rated as low quality (15.3%). Quality assessment results were used to support interpretation of findings and were not applied as a criterion for study exclusion.

References for Quality Assessment Tools

1. Higgins, J. P. T., Altman, D. G., Gøtzsche, P. C., Jüni, P., Moher, D., Oxman, A. D., Savović, J., Schulz, K. F., Weeks, L., & Sterne, J. A. C. (2011). The Cochrane Collaboration's tool for assessing risk of bias in randomised trials. BMJ, 343, d5928. https://doi.org/10.1136/bmj.d5928
2. Wells, G. A., Shea, B., O'Connell, D., Peterson, J., Welch, V., Losos, M., & Tugwell, P. (2000). The Newcastle-Ottawa Scale (NOS) for assessing the quality of nonrandomised studies in meta-analyses. Ottawa Hospital Research Institute. http://www.ohri.ca/programs/clinical_epidemiology/oxford.asp
3. Peters, M. D. J., Marnie, C., Tricco, A. C., Pollock, D., Munn, Z., Alexander, L., McInerney, P., Godfrey, C. M., & Khalil, H. (2020). Updated methodological guidance for the conduct of scoping reviews. JBI Evidence Synthesis, 18(10), 2119–2126. https://doi.org/10.11124/JBIES-20-00167
